# Genome-wide analysis of basic helix–loop–helix superfamily members related to anthocyanin biosynthesis in eggplant (*Solanum melongena* L.)

**DOI:** 10.7717/peerj.7768

**Published:** 2019-10-09

**Authors:** Shiyu Tian, Lujun Li, Min Wei, Fengjuan Yang

**Affiliations:** 1 College of Horticulture Science and Engineering, Shandong Agricultural University/ State Key Laboratory of Crop Biology, Taian, China; 2 Shandong Collaborative Innovation Center of Fruit & Vegetable Quality and Efficient Production, Shandong Agricultural University, Taian, China; 3 Key Laboratory of Biology and Genetic Improvement of Horticultural Crops (Huanghuai Region), Ministry of Agriculture, Taian, China

**Keywords:** *Solanum melongena*, Expression profile, bHLH, Genome-wide, Anthocyanin biosynthesis

## Abstract

The basic helix–loop–helix (bHLH) superfamily is considered the second largest transcription factor (TF) family. It plays regulatory roles in the developmental processes of plants and in their defense responses. In recent years, many bHLH superfamily genes have been identified and characterized in herbaceous and woody plants. However, the comprehensive genomic and functional analyses of these genes in eggplant (*Solanum melongena* L.) have not been reported. In this study, 121 bHLH TFs were identified in the recently released eggplant genome. The phylogeny, gene structure and conserved motifs of the *SmbHLH* gene were comprehensively studied. Subsequently, the phylogenetic relationship between the bHLH of eggplant and the bHLH of other species was analyzed, and the proteins were classified into 17 subfamilies. Among these protein sequences, 16 subgroups were clustered into the functional clades of* Arabidopsis*. Two candidate genes (*SmbHLH1*, *SmbHLH117*) that may be involved in anthocyanin biosynthesis were screened. The tissue specificity or differential expression of the *bHLH* genes in different tissues and under various light and temperature conditions suggested the differential regulation of tissue development and metabolism. This study not only provides a solid foundation for the functional dissection of the eggplant *bHLH* gene family but may also be useful for the future synthesis of anthocyanins in eggplant.

## Introduction

Basic/helix–loop–helix (bHLH) transcription factors (TFs) are widely found in animals and plants (Xu et al., 2017a; Xu et al., 2017b). The bHLH TF is approximately 50–60 amino acids in length and consists of two conserved motifs: the basic region located at the N-terminus and the helix–loop–helix region (HLH region) at the C-terminus (Toledo-Ortiz, Huq & Quail, 2003; Li et al., 2006). The basic region contains approximately 10-15 amino acids, six of which are basic amino acid residues involved in DNA binding. The HLH region is mainly composed of hydrophobic residues and participates in the formation of dimers (Meier-Andrejszki et al., 2007). Aside from the two conserved regions, the remaining bHLH protein sequences differ greatly (Morgenstern & Atchley, 1999). With the emergence of genomic sequencing, *bHLH* superfamily genes such as *Arabidopsis* (Carretero-Paulet et al., 2010), tomato (Sun, Fan & Ling, 2015), apple (Mao et al., 2017), peach (Zhang et al., 2018), and potato (Wang et al., 2018a) have been identified, analyzed, and divided into 15-26 subfamilies on the basis of bioinformatics.

Many studies have shown that bHLH protein was involved in anthocyanin biosynthesis (Nakatsuka et al., 2008; Zhao et al., 2017; Hu et al., 2016) and affected biotic and abiotic stresses (Naing et al., 2018), such as response to light (Zhang et al., 2018), cold (Yao et al., 2018), and hormonal signals (Liu et al., 2018). It also has been shown to participate in organ development (Carretero-Paulet et al., 2010). Anthocyanin synthesis is regulated by MYB-bHLH-WD40 complexes, and the co-regulation of MYB and bHLH has been extensively studied (Xu et al., 2017a; Xu et al., 2017b). *DhMYB2* interacts with *DhbHLH1* to regulate anthocyanin production in *Dendrobium* hybrid petals, and *DhbHLH1* is also responsible for the distinct anthocyanin pigmentation in lip tissues (Li et al., 2017). *VvbHLH003*, *VvbHLH007*, and *VvbHLH010* were found to be related to anthocyanin or flavonol biosynthesis in grapes. The promoters of most genes that are involved in flavonoid or anthocyanin biosynthesis contain a G-box or E-box element that could be recognized by bHLH family members (Wang et al., 2018a; Wang et al., 2018b).

Eggplant (*Solanum melongena* L.) is an important *Solanaceae* crop that is widely cultivated throughout the world (Azuma et al., 2008). There are various colors of the peel of an eggplant such as white, purple, green and orange, which makes this plant a good candidate for studying anthocyanin synthesis. Purple eggplants reportedly have a higher anthocyanin concentration than other dark fruits and vegetables; the concentration in purple eggplants is 2.34 times higher than that of grapes and 7.08 times higher than that of red onions (Wu et al., 2006). In addition, the publication of the eggplant genome data provides a valuable resource for the genome-wide analysis of the bHLH family (Hirakawa et al., 2014). Although many bHLHs have been identified and characterized in numerous plants, the bioinformatics of *bHLH* genes and their function in the anthocyanin synthesis of eggplant have not been reported. In this study, we examined the putative *bHLH* gene subfamily and identified 121 members encoding bHLH TFs. The phylogenetic analyses among the 121 bHLH proteins in eggplant, 152 *Arabidopsis* proteins, and 14 proteins related to anthocyanin synthesis were obtained form 1 tomato protein (Qiu et al., 2016), 1 potato protein (Vincenzo et al., 2014), 2 grape proteins (Hichri et al., 2010), 2 apple proteins (Xie et al., 2012; Xu et al., 2017a), 2 tobacco proteins (Bai et al., 2011), 1 snapdragon protein (Shen et al., 1998), 3 petunia proteins (Shimada, Otsuki & Sakuta, 2007; Gerats et al., 1984), 1 rice gene (Sweeney et al., 2006), and 1 maize protein (Burdo et al., 2014). In addition, the expression profile of *SmbHLHs*, which may be involved in the anthocyanin biosynthesis during fruit development, and the response to LED lights and temperature were also analyzed. These findings provided the first insights into the possible mechanisms of the bHLH proteins in the diversification of plant forms by analyzing the entire bHLH family as well as providing insight into the possible mechanisms of anthocyanin biosynthesis in eggplant.

## Materials and Methods

### Identification and analysis of eggplant bHLH family gene

To identify the bHLH sequence in the eggplant genome, the amino acid data of the eggplant genome was downloaded from the Eggplant Genome Database (http://eggplant.kazusa.or.jp/). A total of 152 *Arabidopsis* bHLH protein sequences were obtained from TAIR (https://www.arabidopsis.org/). In addition, the Hidden Markov Model profile for the bHLH binding domain (PF00010) was downloaded from the Pfam database (http://pfam.xfam.org/). The HMMER program was used to search for bHLH proteins in all eggplant proteins with a cut off *E*-value of 1e^−5^ using PF00010 as a query. Using the *Arabidopsis* bHLH protein sequences as the query sequence, the Blast-p program was used to search the amino acid database in the eggplant genome. Amino acid sequences of the candidate genes for the bHLH family were then obtained. To ensure that the candidate genes obtained were SmbHLH sequences, the obtained candidate sequences were placed in the Pfam (http://pfam.xfam.org/) and SMART databases (http://smart.emblheidelberg.de/) for confirmation. The absence of the bHLH domain was excluded.

### Phylogenetic tree analysis, gene structure and conserved motif characterization

The complete sequences of the amino acids were aligned using MAFFT, and an unrooted phylogenetic tree was constructed using MEGA6 (Tamura et al., 2013) with the following parameters: number of bootstrap replications was 1000, model or method was P-distance, gaps or missing data treatment was pairwise deletion, and only bootstrap values greater than 50 could be displayed on the tree. The full-length gene and CDS sequences of eggplant bHLH genes were downloaded from the Eggplant Genome Website (http://eggplant.kazusa.or.jp/) to form the desired format. The online gene structure display server (http://gsds.cbi.pku.edu.cn/) was used to analyze the bHLH gene structure of the eggplant and the numbers of introns of the genes were clearly obtained. The eggplant bHLH amino acid sequences were downloaded and arranged in the desired order. The bHLH protein sequences of eggplant were uploaded to the online search tool MEME (http://meme-suite.org/tools/meme), and the protein’s conserved motif characteristics were analyzed.

### Expression analysis

Eggplant (*Solanum melongena* L). cv. ‘Jingqiejingang’, ‘Changza NO.8’ (labeled PP, purple peel eggplant), ‘Baiqiezi101’ (labeled WP, white peel eggplant), ‘Lvyichangqie’ (labeled GP, green peel eggplant), and ‘Africa Red Eggplant’ (labeled O-RP, orange-red peel eggplant) seeds were placed on a damp filter paper and incubated in a dark 28 °C incubator until germination. The germinated seeds were sown in the greenhouse at Shandong Agricultural University. When the eggplant seedlings were at the three-true-leaf stage, three temperature treatments (28 °C, 4 °C, and 40 °C) were applied according to Wang et al. (2016). The leaves for RNA extractions were harvested at 1, 3, 6, and 12 h after the treatments.

To investigate the expression patterns in different tissues the stems, leaves, petioles, flowers in bloom, and the peels and pulps of the fruit from different developmental stages of four eggplant varieties (PP, WP, GP, and O-RP) were collected simultaneously from 8-week-old plants under natural conditions. In order to determine the response to different light-emitting diode (LED) light expression, LED red:blue light ratio was 1:1, 3:1, 6:1, and 9:1. The treatment was conducted according to the method of Di (2017). The leaves and peels of the fruit from the eggplant cv. ‘Jingqiejingang’ were harvested.

All of the samples collected were frozen immediately in liquid nitrogen and stored at −80 °C until use. To analyze the expression patterns of the *SmbHLH* genes, a qRT-PCR was performed using the qRT-PCR Probe Kit (VAZYME, China) according to the manufacturer’s instructions. The *β*-actin gene (GenBank: jX524155.1) was used as a reference gene. The primers used for qPCR analysis were designed by Primer Premier 5 and are listed in Table S1. The PCR products were sequenced to confirm the specific amplifications.

## Results

### Identification and characterization of eggplant bHLH gene family

To identify putative bHLH proteins in eggplant, 152 bHLH protein sequences of *Arabidopsis* and the bHLH Hidden Markov Model were used. The Pfam and SMART program tests were used to remove redundant proteins. A total of 121 genes in the eggplant genome were identified as putative members of the *SmbHLH* family (designated as *SmbHLH1*-*SmbHLH121*) (Table 1). The gene ontology (GO) analysis of eggplant bHLH genes was performed in Table S2. The GO analysis revealed that SmbHLHs mainly functioned in protein and DNA binding. To further predict the function of these genes, PSORT online software was used to predict subcellular localization. The results showed that the probability of the genes in the nucleus was more than 90.0% (Table 1). The genes’ ID number in the eggplant genome database was used to find the amino acid number, molecular weight, and isoelectric point of the genes. The annotation information revealed that the length of the SmbHLH amino acid ranged from 67 (SmbHLH28) to 796 (SmbHLH18), and the molecular weights ranged from 7.93 kDa (SmbHLH28) to 91.16 kDa (SmbHLH18), which indicated that the *SmbHLH* gene family may have undergone a long historical evolution and participated in different biological processes. The predicted isoelectric point value of SmbHLH proteins was between 4.32 (SmbHLH119) and 10.11 (SmbHLH106) (Table 1).

**Table 1:** Details of the eggplant bHLH gene family.

Gene name	Gene ID	CDS (bp)	AA	MW (KDa)	PI	Subcellular location prediction
*SmbHLH1*	*Sme2.5_00592.1_g00005.1*	,1983	660	72.46	4.87	nuclear
*SmbHLH2*	*Sme2.5_00014.1_g00019.1*	1,434	477	53.22	7.31	cytoplasmic
*SmbHLH3*	*Sme2.5_00029.1_g00003.1*	1,170	389	42.77	4.81	nuclear
*SmbHLH4*	*Sme2.5_00036.1_g00016.1*	1,260	419	46.24	7.65	nuclear
*SmbHLH5*	*Sme2.5_00047.1_g00028.1*	1,917	638	71.74	9.08	nuclear
*SmbHLH6*	*Sme2.5_00057.1_g00016.1*	1,035	344	38.75	7.14	nuclear
*SmbHLH7*	*Sme2.5_00066.1_g00020.1*	1,320	439	49.23	6.68	nuclear
*SmbHLH8*	*Sme2.5_00081.1_g00022.1*	1,383	460	51.17	6.44	nuclear
*SmbHLH9*	*Sme2.5_00114.1_g00006.1*	1,179	392	44.41	6.47	nuclear
*SmbHLH10*	*Sme2.5_00114.1_g00007.1*	1,101	366	41.20	5.46	nuclear
*SmbHLH11*	*Sme2.5_00130.1_g00003.1*	1,365	454	51.86	6.25	nuclear
*SmbHLH12*	*Sme2.5_00169.1_g00018.1*	2,121	706	77.63	9.43	endoplasmic reticulum
*SmbHLH13*	*Sme2.5_00179.1_g00023.1*	1,758	585	63.76	6.24	nuclear
*SmbHLH14*	*Sme2.5_00186.1_g00003.1*	417	138	15.70	5.22	nuclear
*SmbHLH15*	*Sme2.5_00186.1_g00004.1*	1,335	444	48.86	6.06	nuclear
*SmbHLH16*	*Sme2.5_00190.1_g00012.1*	906	301	33.61	6.70	nuclear
*SmbHLH17*	*Sme2.5_00192.1_g00002.1*	996	331	37.65	5.42	nuclear
*SmbHLH18*	*Sme2.5_00238.1_g00011.1*	2,391	796	91.16	6.64	cytoplasmic
*SmbHLH19*	*Sme2.5_00240.1_g00009.1*	657	218	24.91	8.68	nuclear
*SmbHLH20*	*Sme2.5_00326.1_g00007.1*	1,770	589	64.63	6.76	mitochondrial
*SmbHLH21*	*Sme2.5_00341.1_g00013.1*	795	264	30.75	7.02	nuclear
*SmbHLH22*	*Sme2.5_00386.1_g00001.1*	279	92	10.43	8.53	mitochondrial
*SmbHLH23*	*Sme2.5_00407.1_g00002.1*	918	305	34.90	6.90	nuclear
*SmbHLH24*	*Sme2.5_00407.1_g00003.1*	888	295	32.97	8.45	nuclear
*SmbHLH25*	*Sme2.5_00407.1_g00004.1*	1,071	356	39.82	4.88	nuclear
*SmbHLH26*	*Sme2.5_00411.1_g00009.1*	798	265	30.77	7.58	nuclear
*SmbHLH27*	*Sme2.5_00417.1_g00014.1*	1,038	345	38.78	4.73	nuclear
*SmbHLH28*	*Sme2.5_00475.1_g00013.1*	204	67	7.93	6.97	nuclear
*SmbHLH29*	*Sme2.5_00502.1_g00009.1*	768	255	27.89	8.07	nuclear
*SmbHLH30*	*Sme2.5_00537.1_g00003.1*	1,680	559	61.79	7.93	nuclear
*SmbHLH31*	*Sme2.5_00544.1_g00012.1*	1,611	536	59.64	8.66	cytoplasmic
*SmbHLH32*	*Sme2.5_00563.1_g00003.1*	1,008	335	37.63	6.80	nuclear
*SmbHLH33*	*Sme2.5_00008.1_g00032.1*	1,587	528	57.51	5.25	nuclear
*SmbHLH34*	*Sme2.5_00622.1_g00009.1*	1,269	422	46.39	8.34	nuclear
*SmbHLH35*	*Sme2.5_00622.1_g00011.1*	1,137	378	41.33	9.35	nuclear
*SmbHLH36*	*Sme2.5_00697.1_g00011.1*	1,722	573	64.18	7.56	cytoplasmic
*SmbHLH37*	*Sme2.5_00735.1_g00008.1*	804	267	30.31	6.68	nuclear
*SmbHLH38*	*Sme2.5_00835.1_g00003.1*	729	242	27.28	9.13	nuclear
*SmbHLH39*	*Sme2.5_00925.1_g00014.1*	1,503	500	56.35	6.40	nuclear
*SmbHLH40*	*Sme2.5_01011.1_g00006.1*	867	288	33.14	6.74	nuclear
*SmbHLH41*	*Sme2.5_01047.1_g00014.1*	954	317	35.10	5.37	cytoplasmic
*SmbHLH42*	*Sme2.5_01135.1_g00009.1*	726	241	27.91	8.96	nuclear
*SmbHLH43*	*Sme2.5_01171.1_g00003.1*	1,332	443	51.52	8.96	nuclear
*SmbHLH44*	*Sme2.5_01232.1_g00025.1*	1,185	394	43.65	7.01	nuclear
*SmbHLH45*	*Sme2.5_01286.1_g00009.1*	897	298	33.02	8.83	nuclear
*SmbHLH46*	*Sme2.5_01347.1_g00008.1*	903	300	32.61	5.13	nuclear
*SmbHLH47*	*Sme2.5_01583.1_g00014.1*	1,152	383	41.95	6.85	nuclear
*SmbHLH48*	*Sme2.5_01618.1_g00007.1*	852	283	33.03	6.90	nuclear
*SmbHLH49*	*Sme2.5_01649.1_g00012.1*	750	249	27.80	9.23	nuclear
*SmbHLH50*	*Sme2.5_01725.1_g00003.1*	819	272	30.40	4.69	nuclear
*SmbHLH51*	*Sme2.5_01725.1_g00004.1*	804	267	30.28	6.56	nuclear
*SmbHLH52*	*Sme2.5_01734.1_g00001.1*	906	301	33.87	6.68	nuclear
*SmbHLH53*	*Sme2.5_01764.1_g00004.1*	1,164	387	42.83	6.38	nuclear
*SmbHLH54*	*Sme2.5_01795.1_g00002.1*	2,034	677	73.83	5.45	nuclear
*SmbHLH55*	*Sme2.5_01808.1_g00003.1*	1,569	522	57.24	5.78	nuclear
*SmbHLH56*	*Sme2.5_01944.1_g00011.1*	711	236	26.65	4.99	nuclear
*SmbHLH57*	*Sme2.5_02073.1_g00002.1*	1,317	438	48.38	5.69	cytoplasmic
*SmbHLH58*	*Sme2.5_02104.1_g00004.1*	1,905	634	69.17	5.25	nuclear
*SmbHLH59*	*Sme2.5_02187.1_g00009.1*	603	200	22.41	9.15	nuclear
*SmbHLH60*	*Sme2.5_02242.1_g00002.1*	2,187	728	77.88	7.45	nuclear
*SmbHLH61*	*Sme2.5_02266.1_g00009.1*	1,029	342	37.39	6.67	nuclear
*SmbHLH62*	*Sme2.5_02282.1_g00013.1*	687	228	26.18	6.16	nuclear
*SmbHLH63*	*Sme2.5_02490.1_g00003.1*	1,179	392	43.73	7.93	nuclear
*SmbHLH64*	*Sme2.5_02585.1_g00003.1*	609	202	22.32	5.41	nuclear
*SmbHLH65*	*Sme2.5_02737.1_g00005.1*	465	154	18.22	9.75	nuclear
*SmbHLH66*	*Sme2.5_02756.1_g00007.1*	465	154	17.83	8.43	nuclear
*SmbHLH67*	*Sme2.5_02990.1_g00001.1*	846	281	30.66	9.34	nuclear
*SmbHLH68*	*Sme2.5_03213.1_g00002.1*	1,005	334	37.18	4.90	nuclear
*SmbHLH69*	*Sme2.5_03271.1_g00005.1*	912	303	32.70	5.36	nuclear
*SmbHLH70*	*Sme2.5_03374.1_g00001.1*	756	251	27.66	6.68	nuclear
*SmbHLH71*	*Sme2.5_03403.1_g00001.1*	987	328	36.96	5.18	nuclear
*SmbHLH72*	*Sme2.5_03422.1_g00002.1*	669	222	24.70	9.15	nuclear
*SmbHLH73*	*Sme2.5_03511.1_g00006.1*	1,377	458	50.92	6.00	nuclear
*SmbHLH74*	*Sme2.5_03671.1_g00002.1*	1,485	494	54.79	6.06	nuclear
*SmbHLH75*	*Sme2.5_03826.1_g00003.1*	993	330	36.48	4.90	nuclear
*SmbHLH76*	*Sme2.5_03973.1_g00005.1*	852	283	32.44	6.26	nuclear
*SmbHLH77*	*Sme2.5_03973.1_g00006.1*	831	276	31.56	9.75	nuclear
*SmbHLH78*	*Sme2.5_04004.1_g00006.1*	699	232	25.66	9.40	nuclear
*SmbHLH79*	*Sme2.5_04079.1_g00005.1*	912	303	32.06	5.97	nuclear
*SmbHLH80*	*Sme2.5_04102.1_g00014.1*	1,077	358	39.19	6.83	nuclear
*SmbHLH81*	*Sme2.5_04359.1_g00004.1*	372	123	14.30	6.27	nuclear
*SmbHLH82*	*Sme2.5_04383.1_g00001.1*	906	301	33.64	7.19	nuclear
*SmbHLH83*	*Sme2.5_04514.1_g00004.1*	1,164	387	43.74	5.33	nuclear
*SmbHLH84*	*Sme2.5_04783.1_g00003.1*	954	317	36.27	6.12	nuclear
*SmbHLH85*	*Sme2.5_04864.1_g00002.1*	279	92	10.26	8.52	mitochondrial
*SmbHLH86*	*Sme2.5_05033.1_g00003.1*	552	183	21.42	7.15	cytoplasmic
*SmbHLH87*	*Sme2.5_05101.1_g00002.1*	573	190	20.84	8.11	nuclear
*SmbHLH88*	*Sme2.5_05369.1_g00003.1*	876	291	31.04	5.23	nuclear
*SmbHLH89*	*Sme2.5_05426.1_g00001.1*	1,011	336	37.45	7.33	nuclear
*SmbHLH90*	*Sme2.5_05447.1_g00007.1*	624	207	23.94	8.83	nuclear
*SmbHLH91*	*Sme2.5_05639.1_g00001.1*	864	288	32.98	4.80	nuclear
*SmbHLH92*	*Sme2.5_05849.1_g00001.1*	1,122	373	41.17	7.06	nuclear
*SmbHLH93*	*Sme2.5_06085.1_g00003.1*	1,095	364	41.01	7.77	nuclear
*SmbHLH94*	*Sme2.5_06123.1_g00003.1*	849	282	31.79	6.77	nuclear
*SmbHLH95*	*Sme2.5_06129.1_g00005.1*	969	322	36.04	4.82	nuclear
*SmbHLH96*	*Sme2.5_06337.1_g00004.1*	948	315	34.91	5.99	nuclear
*SmbHLH97*	*Sme2.5_06398.1_g00001.1*	1,524	507	54.75	5.69	nuclear
*SmbHLH98*	*Sme2.5_06460.1_g00003.1*	684	227	25.90	8.41	nuclear
*SmbHLH99*	*Sme2.5_06479.1_g00003.1*	948	315	35.09	6.60	nuclear
*SmbHLH100*	*Sme2.5_06605.1_g00003.1*	693	230	25.79	6.05	nuclear
*SmbHLH101*	*Sme2.5_06668.1_g00002.1*	636	211	24.03	8.48	nuclear
*SmbHLH102*	*Sme2.5_07215.1_g00003.1*	1,611	536	59.94	6.38	nuclear
*SmbHLH103*	*Sme2.5_07421.1_g00001.1*	1,971	656	71.43	7.10	nuclear
*SmbHLH104*	*Sme2.5_07550.1_g00004.1*	732	243	27.46	8.58	nuclear
*SmbHLH105*	*Sme2.5_07791.1_g00001.1*	1,581	526	58.53	8.01	nuclear
*SmbHLH106*	*Sme2.5_09229.1_g00001.1*	279	92	10.35	10.11	mitochondrial
*SmbHLH107*	*Sme2.5_09326.1_g00003.1*	756	251	28.06	8.70	nuclear
*SmbHLH108*	*Sme2.5_09616.1_g00001.1*	786	261	28.41	7.15	nuclear
*SmbHLH109*	*Sme2.5_09905.1_g00002.1*	1,284	427	48.96	9.16	nuclear
*SmbHLH110*	*Sme2.5_10149.1_g00003.1*	1,317	438	49.18	7.38	nuclear
*SmbHLH111*	*Sme2.5_10236.1_g00005.1*	1,338	445	49.12	5.39	nuclear
*SmbHLH112*	*Sme2.5_11033.1_g00001.1*	999	332	37.73	4.81	nuclear
*SmbHLH113*	*Sme2.5_11653.1_g00001.1*	1,377	458	51.73	8.88	nuclear
*SmbHLH114*	*Sme2.5_11931.1_g00001.1*	1,131	377	41.49	5.27	nuclear
*SmbHLH115*	*Sme2.5_12132.1_g00002.1*	699	232	25.73	10.03	nuclear
*SmbHLH116*	*Sme2.5_12177.1_g00001.1*	1,089	362	40.62	4.63	nuclear
*SmbHLH117*	*Sme2.5_12406.1_g00003.1*	1,983	660	74.84	5.91	nuclear
*SmbHLH118*	*Sme2.5_13527.1_g00001.1*	1,392	463	48.19	6.30	nuclear
*SmbHLH119*	*Sme2.5_13712.1_g00001.1*	1,035	344	38.51	4.32	nuclear
*SmbHLH120*	*Sme2.5_13814.1_g00002.1*	471	156	18.26	9.94	nuclear
*SmbHLH121*	*Sme2.5_15542.1_g00001.1*	723	240	28.19	5.44	nuclear

### Gene structure and conserved motif analysis of the eggplant bHLH family

**Figure 1:**
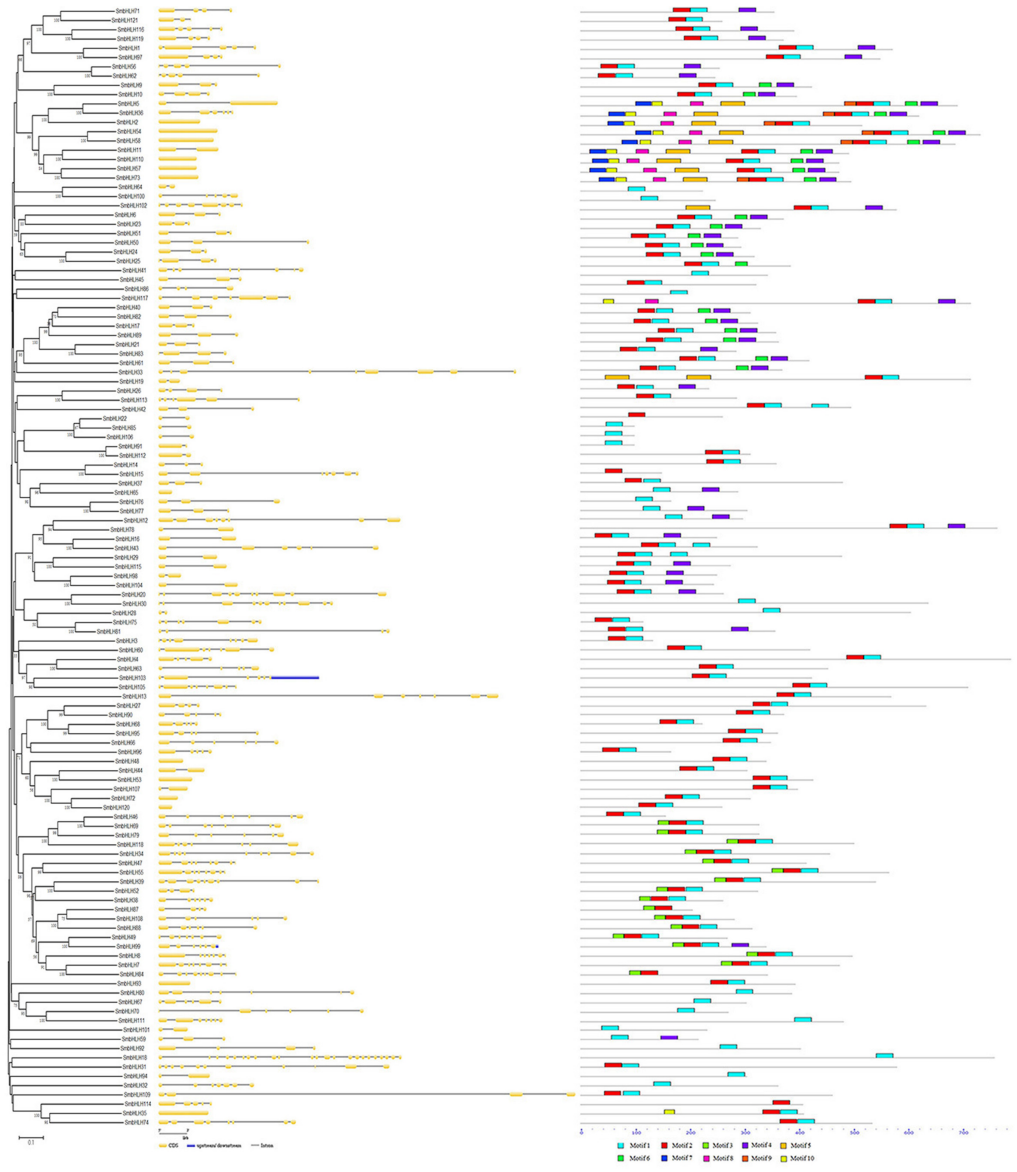
The phylogenetic tree, gene structure and conserved motif analysis of the eggplant bHLH family. On the left is an unroot adjacency phylogenetic tree constructed with 121 SmbHLH amino acid sequences. In the middle is the intron exon map of the *SmbHLH* gene, yellow is an exon and black thin lines represent an intron, and the upstream and downstream region genes of * SmbHLH* are indicated in blue. On the right is the conserved motif of the SmbHLH protein, and the 10 predicted motifs are represented by different colors.

A neighbor-joining phylogenetic tree was constructed with MEGA6 (Fig. 1). The *SmbHLH* genomic sequence and corresponding cDNA sequence of the same *SmbHLH* gene were submitted to GSDS together to show the gene structure. The numbers of introns ranged from 0 to 22 (Fig. 1). In addition, Table S3 shows that 120 genes (99.2%) were below 10, and 13 genes (10.7%) were without introns, 20 genes (16.5%) contained one intron, and the remaining genes contained two or more introns.

The MEME program was used to identify the conserved motif of SmbHLH proteins (Fig. 1). Ten conserved motifs were identified and the protein-conserved motifs of SmbHLH ranged from one to nine. Twenty-three genes (19.0%) had only one conserved motif (Table S3). Each of the predicted motifs were identified only once in each SmbHLH protein sequence. In general, SmbHLH proteins on close adjacent clades of the phylogenetic tree had the same or similar conserved motifs.

### N-terminal conserved domain analysis of eggplant bHLH protein

**Figure 2:**
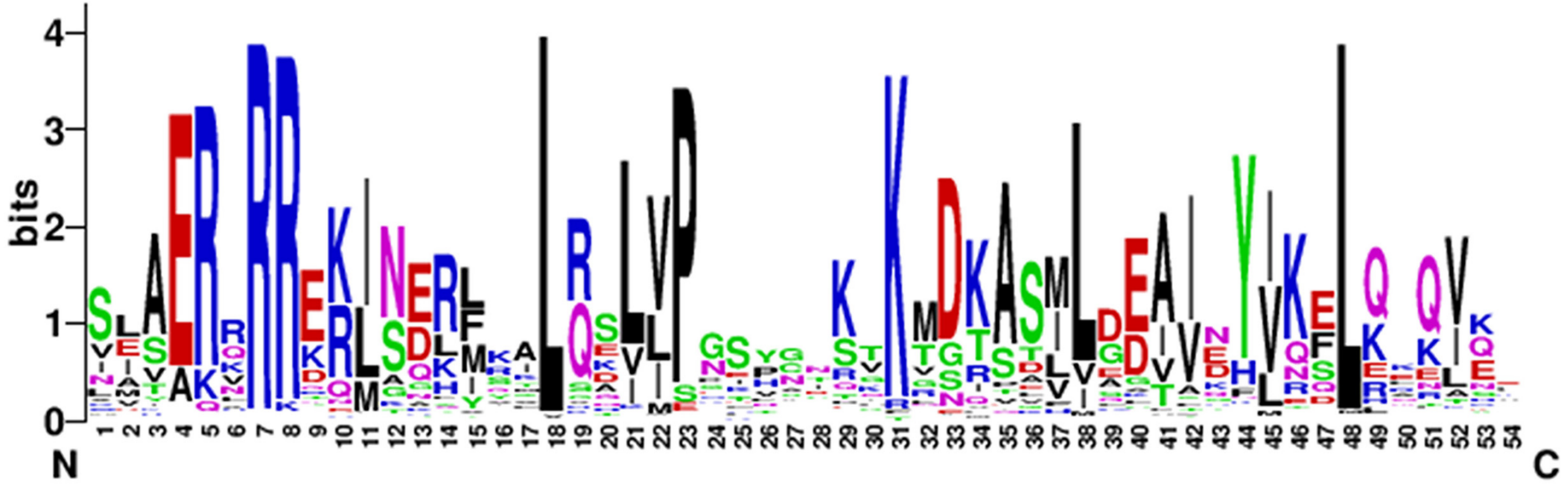
The high conservation of eggplant bHLH protein. Capital letters indicate that the amino acid conserved rate in 121 SmbHLH domains exceeds 50%. The overall height of each stack indicates the conservation of the sequence at that position, whereas the height of letters within each stack represents the relative frequency of the corresponding amino acid.

To analyze the characteristics of the N-terminal DNA-binding region of the bHLH family in eggplant, the amino acid residues-conserved map of bHLH proteins located in the N-terminal were plotted by WEBLOGO (https://weblogo.berkeley.edu/logo.cgi). The conserved motifs of the bHLH proteins in eggplant were [E] -[R] -x(1) -[R] -[R] -x(9) -[L] -x(2) -[L] -x(1) -[P] -x(7) -[K] -x(6) -[L] -x(9)-[L] (Fig. 2).

### Phylogenetic tree analysis of the eggplant bHLH protein family

**Figure 3:**
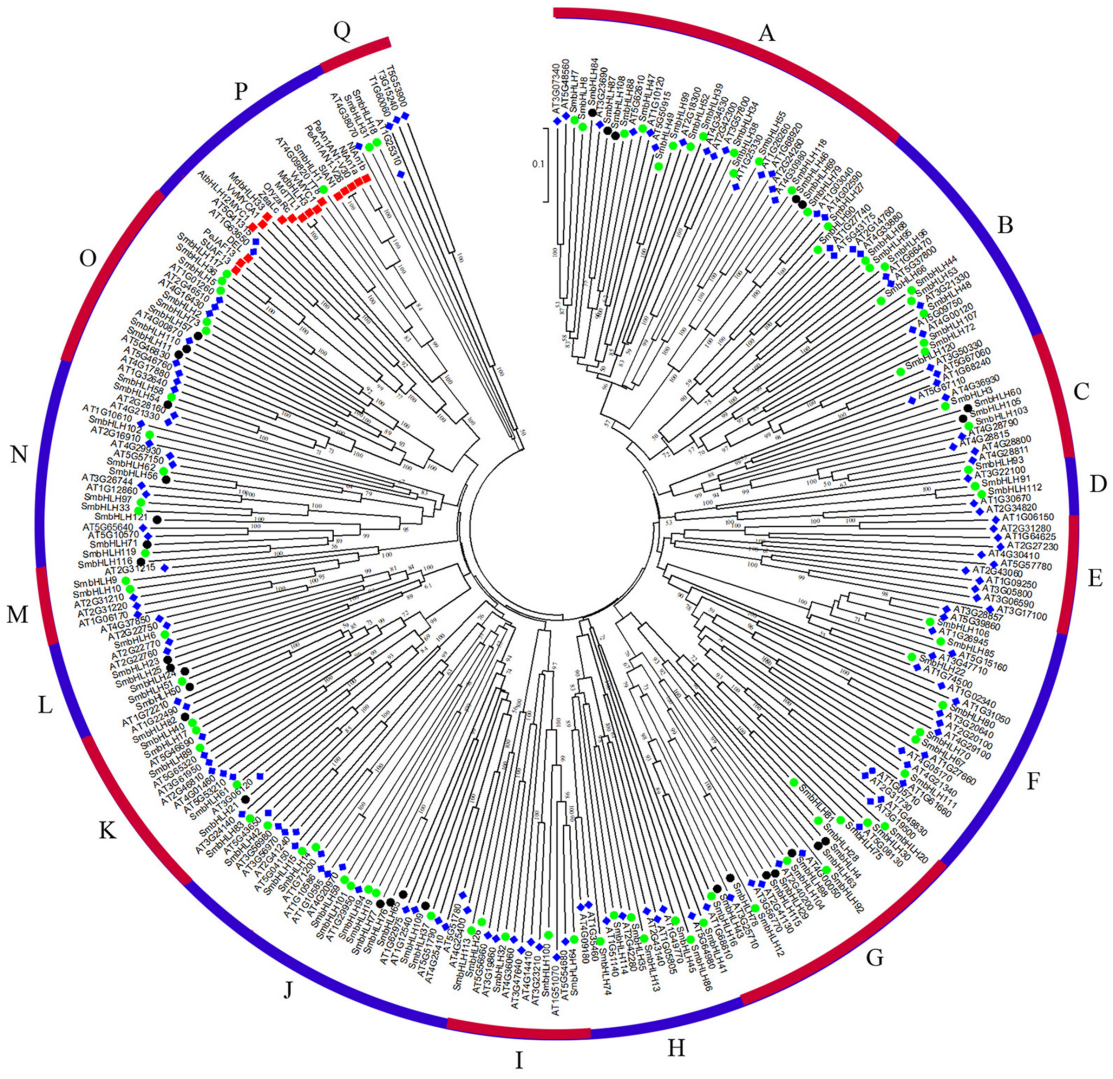
Phylogenetic tree analysis of bHLH between different species. Phylogenetic tree constructed with bHLH of eggplant, *Arabidopsis thaliana* and genes related to anthocyanin biosynthesis including one potato gene, one tomato gene, two grape genes, two apple genes, two tobacco genes, one snapdragon gene, three petunia genes, one rice gene and one maize gene. The green circle represents the eggplant bHLH protein, the blue diamond represents the *Arabidopsis* bHLH protein, and the red box represents the bHLH protein related to anthocyanin synthesis.

To predict the function of the SmbHLH family members of eggplant, an unrooted phylogenetic tree was constructed using 121 SmbHLH proteins, 152 AtbHLH proteins, and 14 proteins related to anthocyanin synthesis, including 1 potato protein, 1 tomato protein, 2 bHLH proteins from grape, 2 apple proteins, 2 tobacco proteins, 1 snapdragon protein, 3 petunia proteins, 1 rice protein, and 1 maize protein. These bHLH proteins could be classified into seventeen distinct subfamilies based on the clade support values and classification from *Arabidopsis* (Fig. 3). Group A was the largest subfamily with 35 proteins, whereas the smallest groups, D and M, contained only six proteins. The numbers of eggplant bHLH proteins within each subfamily varied from 1 to 18. The proteins related to anthocyanin synthesis based on the phylogenetic tree were concentrated in group P. Therefore, the genes involved in anthocyanin synthesis in eggplant were probably *SmbHLH1* and *SmbHLH117*. Further experiments are needed to explore and verify their functions in eggplant.

**Figure 4:**
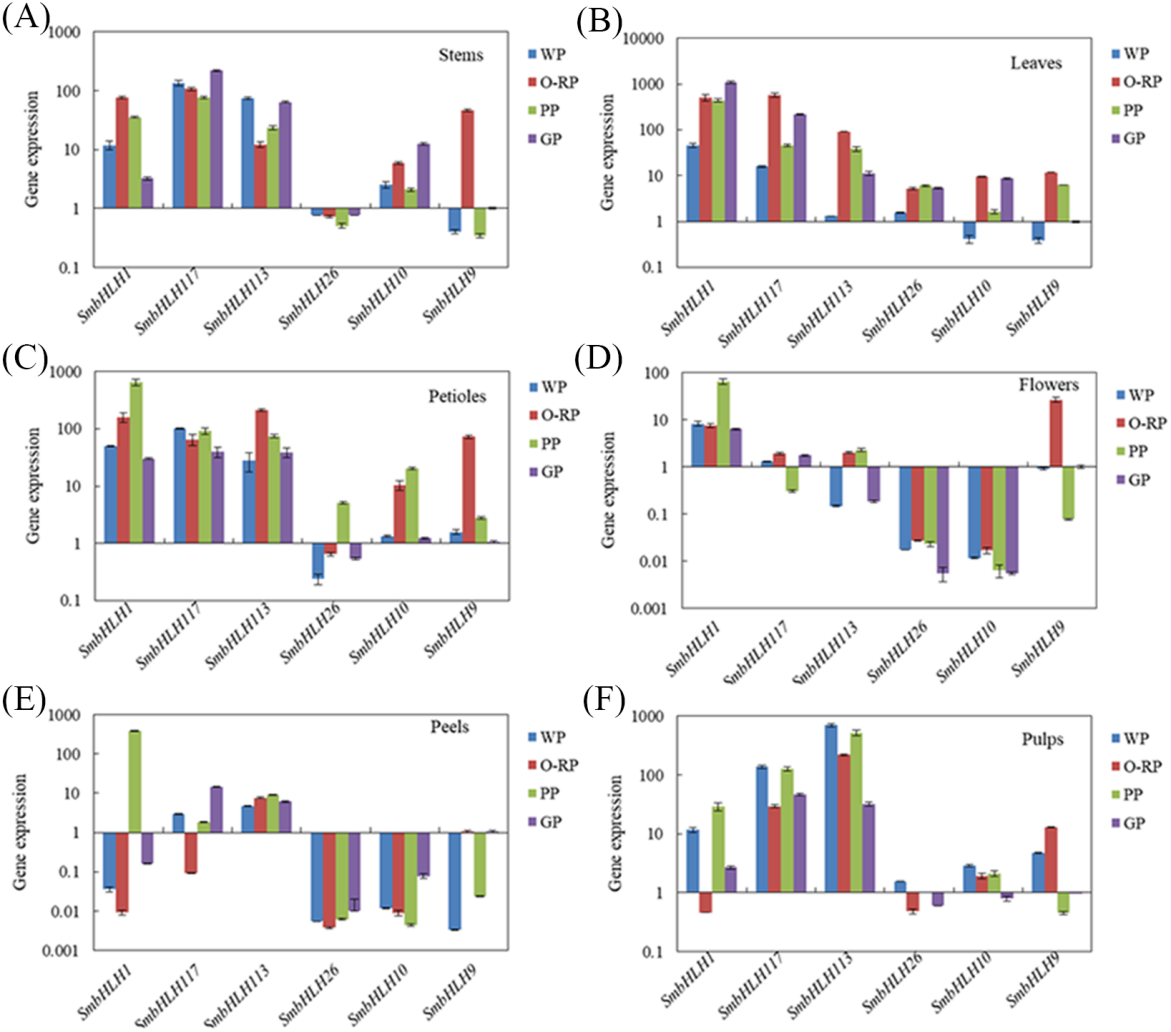
Tissue-specific expression profiles of six *SmbHLH* genes. Relative transcript abundances of *SmbHLH* genes were examined by qRT-PCR. The *y*-axis is the scale of the relative transcript abundance level. The *x*-axis is the number of each *SmbHLH*. The error bars indicate the standard error of three independent repetitions. Total RNA was isolated from stems (A), leaves (B), petioles (C), flowers (D), peels (E) and pulps (F), respectively. The eggplant *β*-actin gene (GenBank JX524155.1) was performed as an internal control. The PCR primers were designed to avoid the conserved region and to amplify products of 150 to 300 bp. Primer sequences were shown in detail in Table S1.

### Expression profiles of eggplant bHLH genes in different colored tissues

Based on the phylogenetic tree (Fig. 3) and the results of previous studies in our laboratory, the genes of *SmbHLH113*, *SmbHLH26*, *SmbHLH9* and *SmbHLH10* were probably involved in anthocyanin biosynthesis. Thus, in order to gain insights into their roles in anthocyanin biosynthesis in differently colored tissues in eggplants, the expression profiles of six putative *SmbHLH* genes, namely, *SmbHLH1*, *SmbHLH117*, *SmbHLH113*, *SmbHLH26*, *SmbHLH9*, and *SmbHLH10* were completed by qRT-PCR. As shown in Fig. 4, the six *SmbHLH* genes showed different patterns of tissue-specific expression in the different peel color varieties of eggplant. The transcript abundance of *SmbHLH1* was higher in the purple tissues of the purple peel eggplant (PP) such as leaves (Fig. 4B), petioles (Fig. 4C), flowers (Fig. 4D) and peels (Fig. 4E), and in the leaves of orange-red peel eggplant (O-RP) (Fig. 4B) and green peel eggplant (GP) (Fig. 4E). However, it was lower in the white and green tissues, such as in the pulps of the four varieties (Fig. 4F), the stems of white peel (WP) and GP eggplant (Fig. 4A), and in the peels of WP, O-RP, and GP (Fig. 4E). Meanwhile, the transcript abundance of *SmbHLH117* was higher in green tissues, such as the stems of GP (Fig. 4A), and the leaves of O-RP and GP (Fig. 4B), but lower in the flowers and peels of the four varieties. *SmbHLH113* expression was higher in the stems of WP and GP (Fig. 4A), the leaves of O-RP and PP (Fig. 4B), the petioles of O-RP (Fig. 4C), and the pulps of WP, O-RP, and PP (Fig. 4F). The transcript abundance of *SmbHLH9* was higher only in the stems, petioles, and flowers of O-RP (Figs. 4A, 4C, 4D). Almost no expression of *SmbHLH26* and *SmbHLH10* were observed in all tissues of the four varieties.

### Expression profiles of eggplant bHLH genes under different LED lights

**Figure 5:**
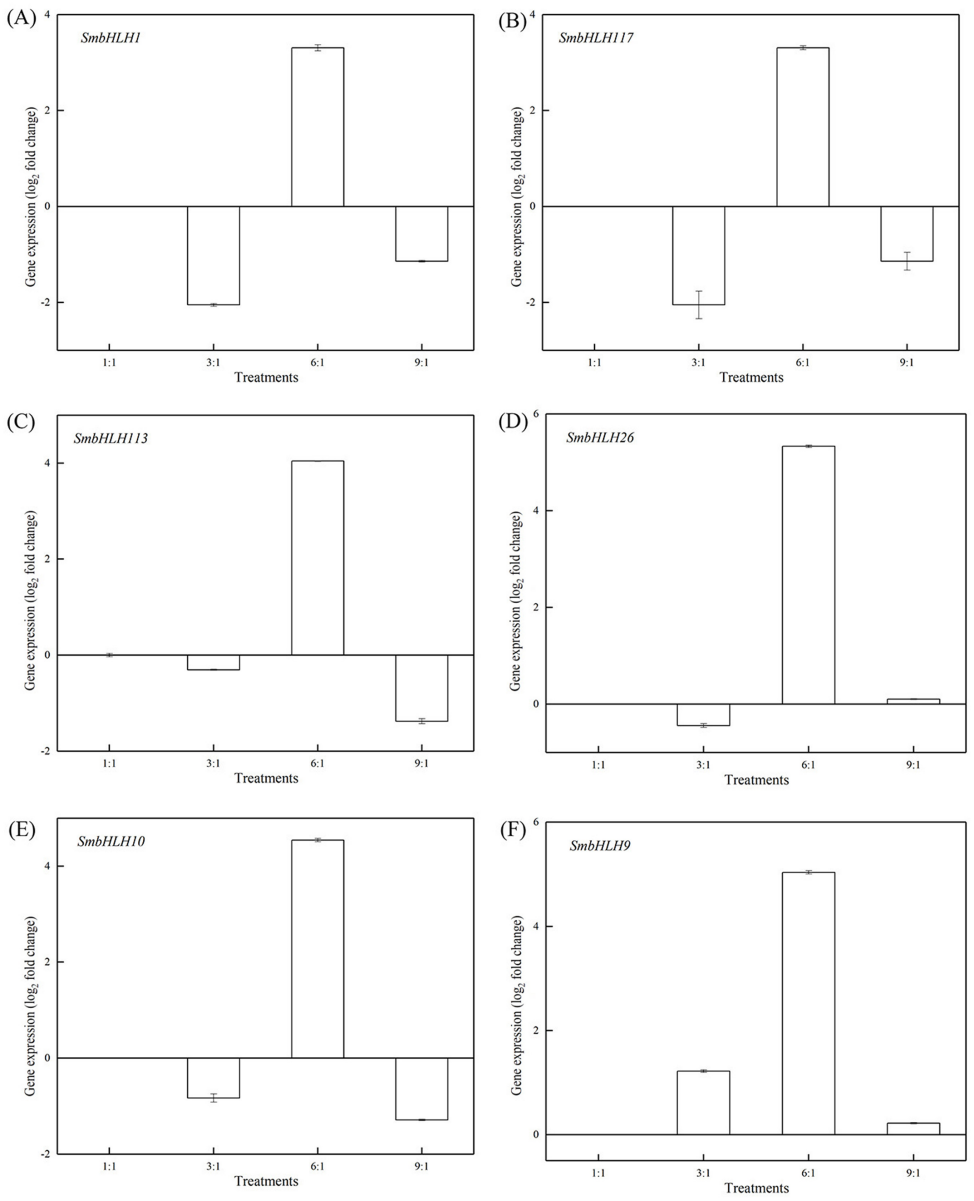
Expression profiles of six *SmbHLH* genes under different LED light (red and blue light ratios of 1:1, 3:1, 6:1, 9:1, respectively) in leaves of eggplant. The *y*-axis is the scale of the relative transcript abundance level. The *x*-axis is the diverse light treatments. (A) Expression profile of the *SmbHLH1* gene under different LED light. (B) Expression profile of the *SmbHLH117* gene under different LED light. (C) Expression profile of the *SmbHLH113* gene under different LED light. (D) Expression profile of the *SmbHLH26* gene under different LED light. (E) Expression profile of the *SmbHLH10* gene under different LED light. (F) Expression profile of the SmbHLH9 gene under different LED light.

**Figure 6:**
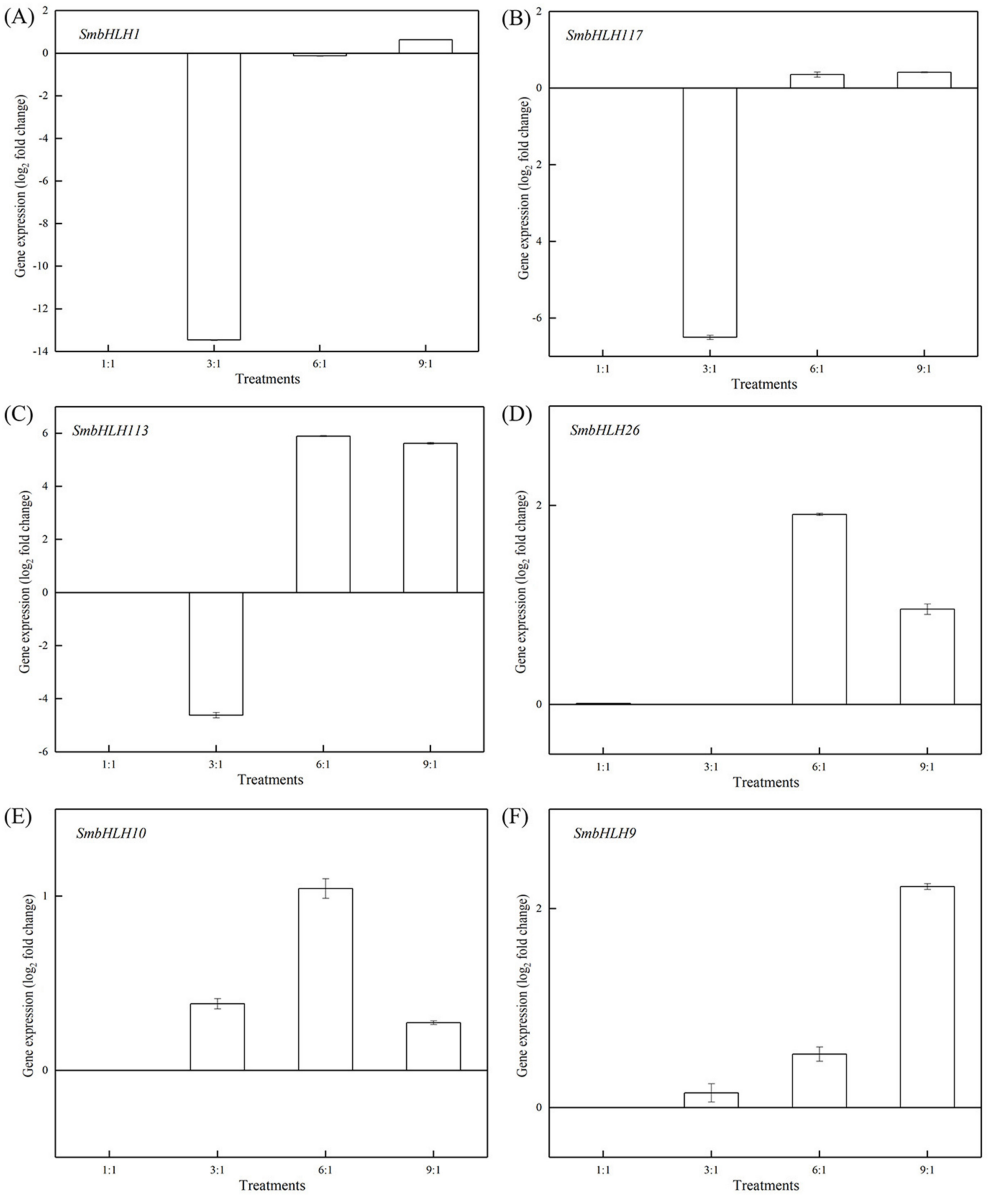
Expression profiles of six *SmbHLH* genes under different LED light (red and blue light ratios of 1:1, 3:1, 6:1, 9:1, respectively) in peels of eggplant. The *y*-axis is the scale of the relative transcript abundance level. The *x*-axis is the diverse light treatments. (A) Expression profile of the *SmbHLH1* gene under different LED light. (B) Expression profile of the *SmbHLH117* gene under different LED light. (C) Expression profile of the *SmbHLH113* gene under different LED light. (D) Expression profile of the *SmbHLH26* gene under different LED light. (E) Expression profile of the *SmbHLH10* gene under different LED light. (F) Expression profile of the SmbHLH9 gene under different LED light.

Light intensity and light quality have different effects on the expression of genes. Under different LED lights (red and blue light ratios of 1:1, 3:1, 6:1, 9:1, respectively), we examined the expressions of the above six *SmbHLH* genes in the leaves (Fig. 5) and peels (Fig. 6) of eggplant. The transcript abundances of *SmbHLH1*, *SmbHLH117*, *SmbHLH113*, *SmbHLH26*, *SmbHLH10*, and *SmbHLH9* genes in leaves (Fig. 5), and *SmbHLH113*, *SmbHLH26*, and *SmbHLH10* in peels of eggplant under R:B = 6:1 treatment (Figs. 6C, 6D, 6E) were higher than those in other treatments. The expression of *SmbHLH1*, *SmbHLH117*, and *SmbHLH9* in the peels of eggplant (Figs. 6A, 6B, 6F) was higher under R:B = 9:1 treatment. These results suggested that all of the above genes related to anthocyanin biosynthesis could positively regulate plants to respond to LED 6 red:1 blue light ratio.

### Response of eggplant bHLH genes expression to temperature

**Figure 7:**
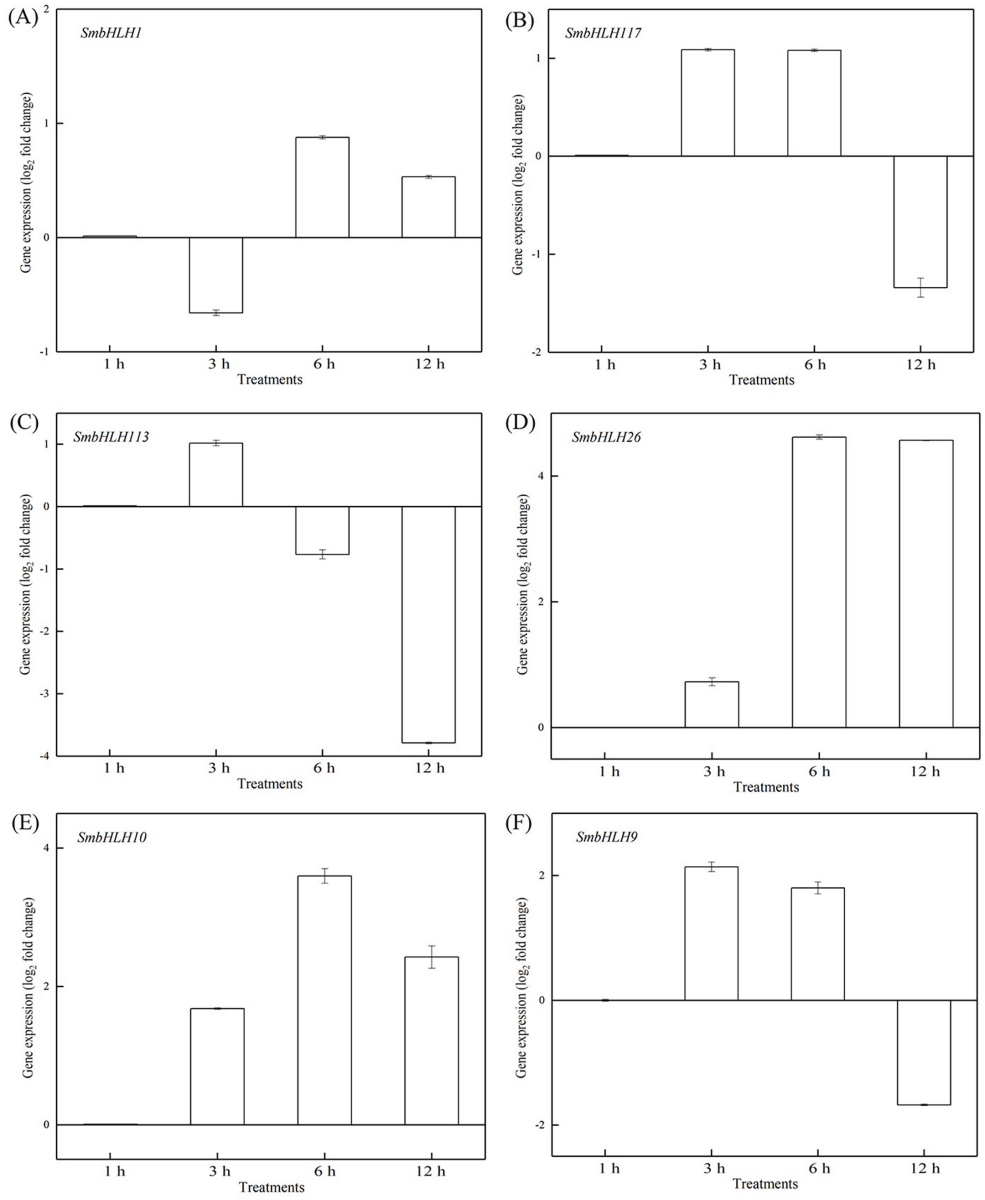
Expression patterns of the six *SmbHLH* genes under optimum temperature (28 °C) treatment. The *y*-axis is the scale of the relative transcript abundance level. The *x*-axis is the time course of 28 °C temperature treatment. (A) Expression profile of the *SmbHLH1* gene under 28 °C treatment. (B) Expression profile of the *SmbHLH117* gene under 28 °C treatment. (C) Expression profile of the *SmbHLH113* gene under 28 °C treatment. (D) Expression profile of the *SmbHLH26* gene under 28 °C treatment. (E) Expression profile of the *SmbHLH10* gene under 28 °C treatment. (F) Expression profile of the SmbHLH9 gene under 28 °C treatment.

**Figure 8:**
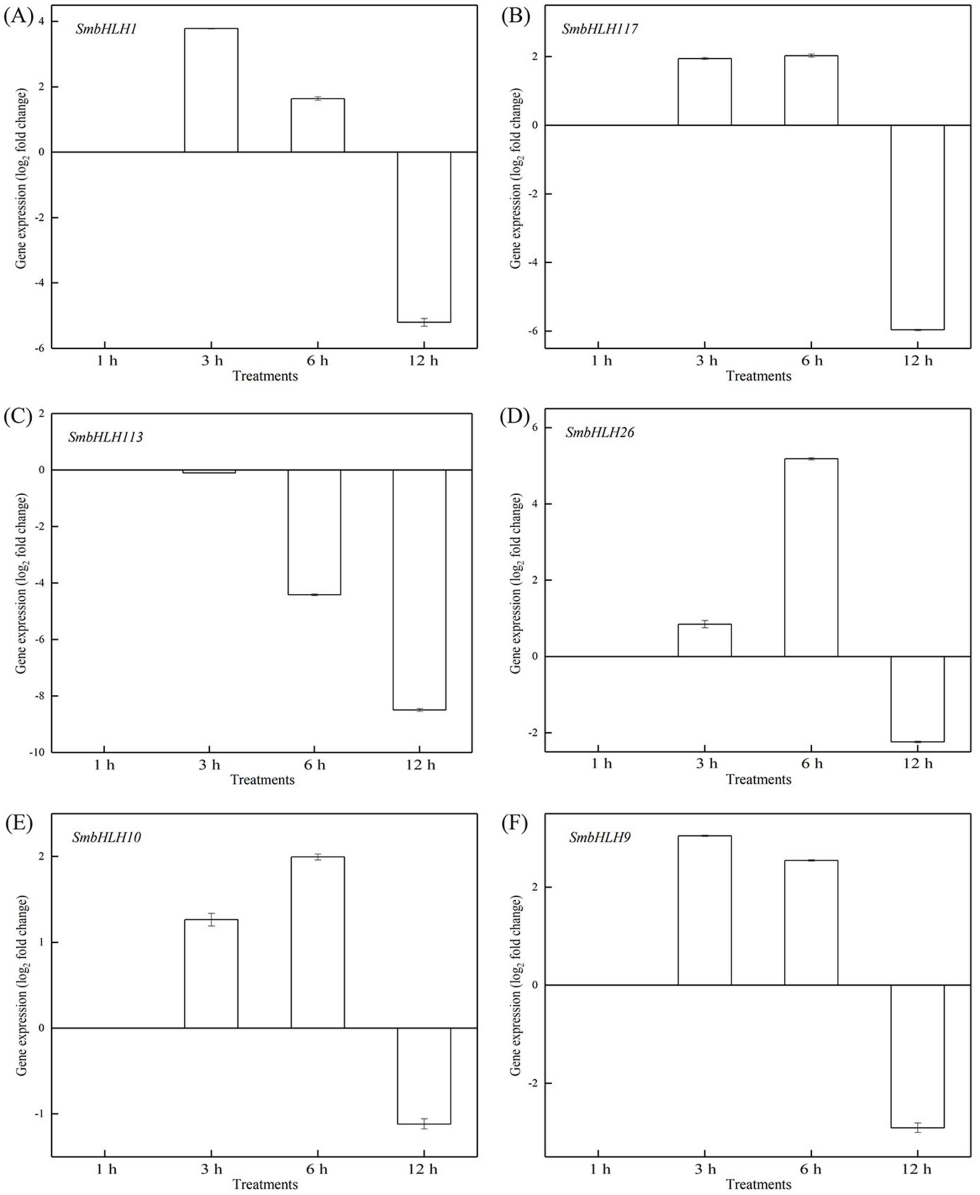
Expression patterns of the six *SmbHLH.* genes under low temperature (4 °C) treatment. The *y*-axis is the scale of the relative transcript abundance level. The *x*-axis is the time course of 4 °C temperature treatment. (A) Expression profile of the *SmbHLH1* gene under 4 °C treatment. (B) Expression profile of the *SmbHLH117* gene under 4 °C treatment. (C) Expression profile of the *SmbHLH113* gene under 4 °C treatment. (D) Expression profile of the *SmbHLH26* gene under 4 °C treatment. (E) Expression profile of the *SmbHLH10* gene under 4 °C treatment. (F) Expression profile of the SmbHLH9 gene under 4 °C treatment.

**Figure 9:**
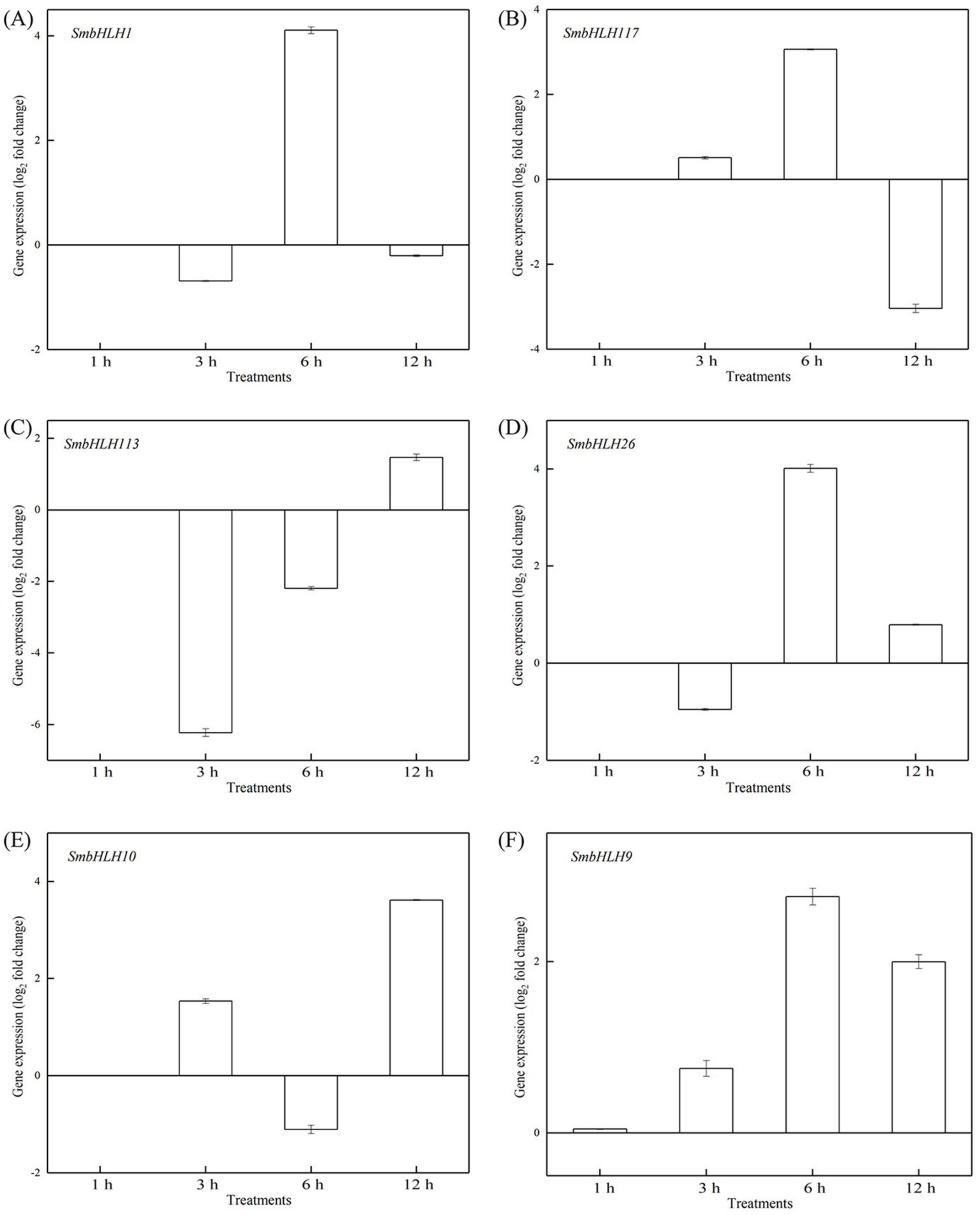
Expression patterns of the six *SmbHLH* genes under high temperature (40 °C) treatment. The *y*-axis is the scale of the relative transcript abundance level. The *x*-axis is the time course of 40 °C temperature treatment. (A) Expression profile of the *SmbHLH1* gene under 40 °C treatment. (B) Expression profile of the *SmbHLH117* gene under 40 °C treatment. (C) Expression profile of the *SmbHLH113* gene under 40 °C treatment. (D) Expression profile of the *SmbHLH26* gene under 40 °C treatment. (E) Expression profile of the *SmbHLH10* gene under 40 °C treatment. (F) Expression profile of the SmbHLH9 gene under 40 °C treatment.

To investigate the roles of *SmbHLH* genes under different temperatures, trials at 28 °C (optimum temperature), 4 °C (low temperature), and 40 °C (high temperature) were conducted. The relative expression levels of the above six genes, namely, *SmbHLH1*, *SmbHLH117*, *SmbHLH113*, *SmbHLH26*, *SmbHLH9*, and *SmbHLH10* were performed by qRT-PCR (Figs. 7–9). At the optimum growth temperature of eggplant at 28°, there were large changes in gene expression with the increase of treatment time. The expression of *SmbHLH26* and *SmbHLH10* peaked at 6 h (Figs. 7D, 7E) and *SmbHLH9* (Fig. 7F) at 3 h, whereas the expression levels of the other genes were relatively stable. Under treatment at 4 °C, the transcript abundance levels of *SmbHLH1*, *SmbHLH117*, and *SmbHLH9* were higher after 3 h of cold treatment (Figs. 8A, 8B, 8F), while *SmbHLH26* and *SmbHLH10* were higher after 6 h of cold treatment (Figs. 8D, 8E). The expression levels of the six genes were greatly induced by a high temperature of 40 °C (Fig. 9). The expression levels of *SmbHLH1*, *SmbHLH117, SmbHLH26,* and *SmbHLH9* peaked at 6 h (Figs. 9A, 9B, 9D, 9F), while *SmbHLH113* and *SmbHLH10* peaked at 12 h (Figs. 9C, 9E). These results suggest that all of the above-mentioned genes that are related to anthocyanin biosynthesis positively regulate the plants response to temperature changes, especially to stress caused by low and high temperatures.

## Discussion

A number of studies have shown that the bHLH TFs may respond to multiple stressors, disease resistance, or growth control in plants (Samira et al., 2018; Cheng et al., 2018). The bHLH family is a key determinant of the specification and differentiation of cells in plants and vertebrates (Carretero-Paulet et al., 2010). A total of 121 genes in the eggplant genome were identified as putative members of the *SmbHLH* family. Subcellular localization predicted that most of the SmbHLH proteins were located in the nucleus (Table 1). The numbers of bHLH proteins in eggplant were similar to those in *Solanaceae* plants, like potato and tomato. 159 *SlbHLH* genes were identified in the tomato genome (Sun, Fan & Ling, 2015) and 124 StbHLH proteins were identified in potato (Wang et al., 2018a).

Toledo-Ortiz, Huq & Quail (2003) found that the residues Ile-20, Leu-24, Gln-28, Lys-36, Met-50, Ile-55, Val-58 and Leu-61 in the bHLH domains of plants were more conserved than in animals. The results of this study are consistent with previous research and the residues Arg-7, Leu-18 and Leu-48 showed extreme conservation among the 121 bHLH proteins of eggplant (Fig. 2). These conserved amino acid residues may play an important role in the evolution of eggplant (Wang et al., 2015). The residues Glu-4, Arg-7 and Arg-8 in the basic region of the bHLH domain play an important role in DNA binding (Atchley & Fitch, 1997), and Leu-18 and Leu-48 in the helix-loop-helix regions play an important role in dimerization activity (Simionato et al., 2007). The basic region of the bHLH proteins contained 8 amino acids in eggplant, which was six amino acids shorter than that described by Atchley (Atchley, Terhalle & Dress, 1999).

To date, the biological functions of most SmbHLHs remain unclear. However, approximately 40% of *Arabidopsis* bHLH proteins have been functionally characterized (Sun, Fan & Ling, 2015). The previous research revealed that the classification characteristics of the bHLH family could be divided into 15–25 subfamilies (Pires & Dolan, 2010). In this study, the phylogenetic tree was constructed with the bHLH domain regions of bHLH proteins of 11 species (121 eggplant proteins, 152 *Arabidopsis* proteins, 1 potato protein, 1 tomato protein, 2 grape proteins, 2 apple proteins, 2 tobacco proteins, 1 snapdragon protein, 3 petunia proteins, 1 rice protein, and 1 maize protein), and these bHLH proteins were classified into seventeen distinct subfamilies (Fig. 3). The classification of these subfamilies is common and consistent with the subfamily classification previously reported in phylogenetic tree analysis in other species (Sun, Fan & Ling, 2015; Zhang et al., 2018; Wang et al., 2018a; Wang et al., 2018b). Members within the same clade may have common evolutionary origins and conserved molecular functions, and may be involved in the same pathway or biological process (Pires & Dolan, 2010). In this study, eggplant *SmbHLH61*, *SmbHLH83* and *SmbHLH21* genes and *Arabidopsis thaliana AT5G53210*, *AT3G06120* and *AT3G24140* genes (Liu et al., 2018; Pillitteri et al., 2007) are clustered in one clade. Therefore, we concluded that the *SmbHLH61*, *SmbHLH83* and *SmbHLH21* genes of eggplant may also regulate the development of stomata in eggplant leaves. Previous studies have shown that the bHLH subfamily plays important roles in anthocyanin synthesis (Zhang et al., 2018; Zhao et al., 2017). The two eggplant bHLH orthologous *SmbHLH1* and *SmbHLH117* genes are clustered in the same subgroup with the genes involved in anthocyanin synthesis, so the eggplant *SmbHLH1* and *SmbHLH117* genes may be involved in regulating the anthocyanin biosynthesis of eggplant. The results showed that the transcript abundance of *SmbHLH1* was higher in the purple tissues of the purple peel eggplant such as leaves, petioles, flowers and peels. However, it was lower in the white and green tissues. Meanwhile, the transcript abundance of *SmbHLH117* was higher in green tissues, but lower in the flowers and peels of the four varieties (Fig. 4).

Light is an inducing factor for anthocyanin synthesis and can increase the content of anthocyanins in most plants (Mancinelli, 1985). The light intensity and light environment have different influences on the synthesis. The light can improve *MdbHLH33* expression levels in apple, and promote the accumulation of anthocyanins in the skin (Takos et al., 2006). In this study, the results suggested that the genes of *SmbHLH1*, *SmbHLH117*, *SmbHLH113*, *SmbHLH26*, *SmbHLH10*, and *SmbHLH9* related to anthocyanin biosynthesis could positively regulate plants to respond to LED 6 red: 1 blue light ratio (Figs. 5 and 6). In addition, anthocyanin synthesis in plants is affected by temperature (Lin-Wang et al., 2011). Low temperature can stimulate the accumulation of anthocyanins by up-regulating the expression of biosynthetic genes (Crifo et al., 2011). Our results showed that all of the above-mentioned genes related to anthocyanin biosynthesis positively responded to temperature stress at 4 °C (Fig. 8) and 40 ° C (Fig. 9).

## Conclusions

A total of 121 *SmbHLH* genes were identified from the eggplant genome and their gene structures and conserved motifs of amino acids were characterized. Phylogenetic comparisons of the *SmbHLH* gene families between eggplant and other species revealed that there were two *SmbHLH* genes (*SmbHLH1* and *SmbHLH117*) related to anthocyanin biosynthesis in eggplant. There were different expression patterns of six *SmbHLH* genes related to anthocyanin biosynthesis in various tissues of different eggplant varieties and under LED light qualities and temperature conditions. These findings provide comprehensive information for further analysis of the biological function and evolution of the *SmbHLH* gene family in eggplant.

##  Supplemental Information

10.7717/peerj.7768/supp-1 Table S1 Primers for real-time quantitative PCR

10.7717/peerj.7768/supp-2 Table S2 Gene Ontology analysis of SmbHLH

10.7717/peerj.7768/supp-3 Table S3 The number of introns and conserved motifs for each gene in the phylogenetic tree of eggplant

10.7717/peerj.7768/supp-4 Supplemental Information 1 The alignment results of the bHLH protein sequences with MAFFT

10.7717/peerj.7768/supp-5 Supplemental Information 2 Sm-HMM Eggplant bHLH proteins using HMMER program search.

10.7717/peerj.7768/supp-6 Supplemental Information 3 The sequences of SmbHLH

10.7717/peerj.7768/supp-7 Supplemental Information 4 Raw data of the expression of genes applied for Fig. 4–Fig. 9

## Additional Information and Declarations

### Data Availability

The following information was supplied regarding data availability: The raw measurements are available in the Supplemental Files.
