# Supplementary material for: Genome-wide analysis of basic helix–loop–helix superfamily members related to anthocyanin biosynthesis in eggplant (Solanum melongena L.)
Source: PeerJ. 2019 Oct 9;7:e7768. doi: 10.7717/peerj.7768 (PMC6790105; doi:10.7717/peerj.7768)
Supplement: Table S1 [file peerj-07-7768-s001.docx]

Table S1 Primers for real-time quantitative PCR

| Primer name | Primer sequence |
| --- | --- |
| *SmbHLH1F* | 5'-GATGATGGCTCTAATAATATGGACTCTG-3' |
| *SmbHLH1R* | 5'-GGCTGATACTGTTTGGGAATAATGG-3' |
| *SmbHLH117F* | 5'-GTGATATGGATGAGATGGAACCTG-3' |
| *SmbHLH117R* | 5'-ATGTTGGAAGACTGAACTGTATGG-3' |
| *SmbHLH113F* | 5'-CCGAGCAGACCATAAGACCAG-3' |
| *SmbHLH113R* | 5'-GAGAGGACGAGGAAGTAGAAGG-3' |
| *SmbHLH26F* | 5'-GAATTTACAAAGCAAACAGAGGAAAC-3' |
| *SmbHLH26R* | 5'-CGAGACGAAGAATGATGAGAAGG-3' |
| *SmbHLH10F* | 5'-GCAACATGAAGGACTAGCAGAAG-3' |
| *SmbHLH10R* | 5'-CACCACCACCACCATCAATG-3' |
| *SmbHLH9F* | 5'-GCAGCAGCAGCAGTTGAATC-3' |
| *SmbHLH9R* | 5'-TGAGGATGATGAAGAAATTGGGTAAC-3' |
| *β-actinF* | 5'-GGTTACTCATTCACCACCACAGC-3' |
| *β-actinR* | 5'-ATAGGACCTCAGGGCAACGG-3' |
