# Supplementary material for: Genome-wide analysis of basic helix–loop–helix superfamily members related to anthocyanin biosynthesis in eggplant (Solanum melongena L.)
Source: PeerJ. 2019 Oct 9;7:e7768. doi: 10.7717/peerj.7768 (PMC6790105; doi:10.7717/peerj.7768)
Supplement: Table S3 [file peerj-07-7768-s003.docx]

Table S3 The number of introns and conserved motifs for each gene in the phylogenetic tree of eggplant

| Gene name | Number of conservative motifs | Number of introns |
| --- | --- | --- |
| *SmbHLH71* | 3 | 3 |
| *SmbHLH121* | 2 | 2 |
| *SmbHLH116* | 3 | 4 |
| *SmbHLH119* | 3 | 3 |
| *SmbHLH33* | 3 | 4 |
| *SmbHLH97* | 3 | 3 |
| *SmbHLH56* | 3 | 4 |
| *SmbHLH62* | 3 | 4 |
| *SmbHLH9* | 4 | 2 |
| *SmbHLH10* | 4 | 3 |
| *SmbHLH5* | 9 | 1 |
| *SmbHLH36* | 9 | 5 |
| *SmbHLH2* | 7 | 0 |
| *SmbHLH54* | 9 | 0 |
| *SmbHLH58* | 9 | 0 |
| *SmbHLH11* | 8 | 1 |
| *SmbHLH110* | 8 | 0 |
| *SmbHLH57* | 8 | 0 |
| *SmbHLH73* | 9 | 0 |
| *SmbHLH64* | 1 | 1 |
| *SmbHLH100* | 1 | 4 |
| *SmbHLH102* | 4 | 7 |
| *SmbHLH6* | 4 | 2 |
| *SmbHLH23* | 4 | 2 |
| *SmbHLH51* | 4 | 2 |
| *SmbHLH50* | 4 | 2 |
| *SmbHLH24* | 4 | 2 |
| *SmbHLH25* | 3 | 3 |
| *SmbHLH41* | 1 | 7 |
| *SmbHLH45* | 2 | 2 |
| *SmbHLH86* | 1 | 3 |
| *SmbHLH117* | 5 | 6 |
| *SmbHLH40* | 4 | 2 |
| *SmbHLH82* | 4 | 2 |
| *SmbHLH17* | 4 | 2 |
| *SmbHLH89* | 4 | 2 |
| *SmbHLH21* | 3 | 2 |
| *SmbHLH83* | 4 | 3 |
| *SmbHLH61* | 4 | 2 |
| *SmbHLH1* | 4 | 8 |
| *SmbHLH19* | 3 | 1 |
| *SmbHLH26* | 2 | 3 |
| *SmbHLH113* | 3 | 6 |
| *SmbHLH42* | 1 | 2 |
| *SmbHLH22* | 1 | 1 |
| *SmbHLH85* | 1 | 1 |
| *SmbHLH106* | 1 | 1 |
| *SmbHLH91* | 2 | 1 |
| *SmbHLH112* | 2 | 1 |
| *SmbHLH14* | 1 | 2 |
| *SmbHLH15* | 2 | 6 |
| *SmbHLH37* | 2 | 2 |
| *SmbHLH65* | 1 | 0 |
| *SmbHLH76* | 2 | 2 |
| *SmbHLH77* | 2 | 2 |
| *SmbHLH12* | 3 | 7 |
| *SmbHLH78* | 3 | 1 |
| *SmbHLH16* | 3 | 1 |
| *SmbHLH43* | 3 | 5 |
| *SmbHLH29* | 3 | 1 |
| *SmbHLH115* | 3 | 1 |
| *SmbHLH98* | 3 | 1 |
| *SmbHLH104* | 3 | 1 |
| *SmbHLH20* | 1 | 9 |
| *SmbHLH30* | 1 | 10 |
| *SmbHLH28* | 1 | 1 |
| *SmbHLH75* | 3 | 6 |
| *SmbHLH81* | 2 | 3 |
| *SmbHLH3* | 2 | 7 |
| *SmbHLH60* | 2 | 6 |
| *SmbHLH4* | 2 | 4 |
| *SmbHLH63* | 2 | 4 |
| *SmbHLH103* | 2 | 5 |
| *SmbHLH105* | 2 | 7 |
| *SmbHLH13* | 2 | 6 |
| *SmbHLH27* | 2 | 3 |
| *SmbHLH90* | 2 | 4 |
| *SmbHLH68* | 2 | 4 |
| *SmbHLH95* | 2 | 4 |
| *SmbHLH66* | 2 | 5 |
| *SmbHLH96* | 2 | 4 |
| *SmbHLH48* | 2 | 0 |
| *SmbHLH44* | 2 | 1 |
| *SmbHLH53* | 2 | 0 |
| *SmbHLH107* | 2 | 1 |
| *SmbHLH72* | 2 | 0 |
| *SmbHLH120* | 2 | 0 |
| *SmbHLH46* | 2 | 6 |
| *SmbHLH69* | 3 | 6 |
| *SmbHLH79* | 3 | 5 |
| *SmbHLH118* | 3 | 6 |
| *SmbHLH34* | 3 | 8 |
| *SmbHLH47* | 3 | 7 |
| *SmbHLH55* | 3 | 7 |
| *SmbHLH39* | 3 | 8 |
| *SmbHLH52* | 3 | 3 |
| *SmbHLH38* | 3 | 5 |
| *SmbHLH87* | 2 | 3 |
| *SmbHLH108* | 3 | 5 |
| *SmbHLH88* | 3 | 5 |
| *SmbHLH49* | 3 | 6 |
| *SmbHLH99* | 4 | 5 |
| *SmbHLH8* | 3 | 6 |
| *SmbHLH7* | 3 | 7 |
| *SmbHLH84* | 2 | 8 |
| *SmbHLH93* | 2 | 0 |
| *SmbHLH80* | 1 | 6 |
| *SmbHLH67* | 1 | 5 |
| *SmbHLH70* | 1 | 5 |
| *SmbHLH111* | 1 | 6 |
| *SmbHLH101* | 1 | 1 |
| *SmbHLH59* | 2 | 2 |
| *SmbHLH92* | 1 | 3 |
| *SmbHLH18* | 1 | 22 |
| *SmbHLH31* | 2 | 10 |
| *SmbHLH94* | 1 | 1 |
| *SmbHLH32* | 1 | 6 |
| *SmbHLH109* | 2 | 3 |
| *SmbHLH114* | 1 | 4 |
| *SmbHLH35* | 3 | 0 |
| *SmbHLH74* | 2 | 7 |
